# Doença de Coronavírus-19 e o Miocárdio

**DOI:** 10.36660/abc.20200373

**Published:** 2020-06-29

**Authors:** José Albuquerque de Figueiredo, Fabiana G. Marcondes-Braga, Lidia Zytinski Moura, André Melo e Silva de Figueiredo, Viviane Melo e Silva de Figueiredo, Ricardo Mourilhe-Rocha, Evandro Tinoco Mesquita

**Affiliations:** 1 Universidade Federal do Maranhão São LuisMA Brasil Universidade Federal do Maranhão, São Luis, MA - Brasil; 2 Hospital das Clínicas Faculdade de Medicina Universidade de São Paulo São PauloSP Brasil Instituto do Coração do Hospital das Clínicas da Faculdade de Medicina da Universidade de São Paulo,São Paulo, SP - Brasil; 3 Pontifícia Universidade Católica do Paraná CuritibaPR Brasil Pontifícia Universidade Católica do Paraná, Curitiba, PR - Brasil; 4 Universidade do Estado do Rio de Janeiro Hospital Pró-cardíaco Brasil Universidade do Estado do Rio de Janeiro e Hospital Pró-cardíaco. Brasil; 5 C.T.E.B UHG Brazil Educador C.T.E.B./UHG.Brazil

**Keywords:** Miocárdio/lesões, Troponina, Doenças Inflamatórias, Miocardite, Síndrome de Takotsubo, Biomarcadores, Coronavirus, COVID-19, Pandemia, Cardiomiopatia Dilatada, Microangiopatias Trombóticas

## Abstract

A infecção pelo coronavírus denominada COVID-19 promoveu crescente interesse de cardiologistas, emergencistas, intensivistas e pesquisadores, pelo estudo do acometimento miocárdico partindo de diferentes formas clínicas decorrentes de desmodulação imunoinflamatória e neuro-humoral.O acometimento miocárdico pode ser mínimo e apenas identificado a partir de alterações eletrocardiográficas, principalmente por aumento de troponinas cardíacas, ou no outro lado do espectro pelas formas de miocardite fulminante e síndrome de takotsubo.A descrição de provável miocardite aguda tem sido comumente apoiada pela observação da troponina elevada em associação com disfunção. A clássica definição de miocardite, respaldada pela biópsia endomiocárdica de infiltrado inflamatório é rara, e foi observada em um único relato de caso até o momento, não se identificando o vírus no interior dos cardiomiócitos.Assim, o fenômeno que se tem documentado é de injúria miocárdica aguda, sendo obrigatório afastar doença coronária obstrutiva a partir da elevação de marcadores de necrose miocárdica, associada ou não à disfunção ventricular, provavelmente associada à tempestade de citoquinas e outros fatores que podem sinergicamente promover lesão miocárdica, tais como hiperativação simpática, hipoxemia, hipotensão arterial e fenômenos trombóticos microvasculares.Fenômenos inflamatórios sistêmicos e miocárdicos após infecção viral estão bem documentados, podendo evoluir para remodelamento cardíaco e disfunção miocárdica. Portanto, será importante a cardiovigilância desses indivíduos para monitorar o desenvolvimento do fenótipo de miocardiopatia dilatada.A presente revisão apresenta os principais achados etiofisiopatológicos, descrição da taxonomia desses tipos de acometimento cardíaco e sua correlação com as principais formas clínicas do componente miocárdico presente nos pacientes na fase aguda de COVID-19.

## Introdução

A lesão miocárdica, evidenciada por biomarcadores cardíacos elevados, foi reconhecida entre os primeiros casos de COVID-19, na China. O relatório do Conselho Nacional de Saúde da China relatou que quase 12% dos pacientes sem doença cardiovascular (DCV) conhecida apresentaram níveis elevados de troponina ou parada cardíaca durante a hospitalização.^1^

Estes achados estimularam a pesquisa e o interesse por parte de cardiologistas, intensivistas e pesquisadores clínicos, pelo reconhecimento precoce destas anormalidades, bem como a busca dos mecanismos fisiopatológicos e o seu real impacto prognóstico.

Ao lado disso, foi identificado que indivíduos portadores de DCVs prévias, apresentavam maior risco para o desenvolvimento das formas graves e maior mortalidade.

Desta forma, entender o espectro do acometimento miocárdico, primário ou secundário, bem como os mecanismos etiofisiopatológicos envolvidos, são de fundamental importância para o desenvolvimento de estratégias terapêuticas, que possam prevenir e atenuar a agressão miocárdica presente na fase aguda.

## SARS-CoV-2 e o mecanismo de agressão celular direta

A infecção por SARS-CoV-2 é causada pela ligação da proteína *spike* da superfície viral ao receptor da enzima conversora de angiotensina 2 (ECA-2) humana após a ativação da proteína *spike* pela protease serina 2 transmembrana (TMPRSS2).

**Figura 1:**
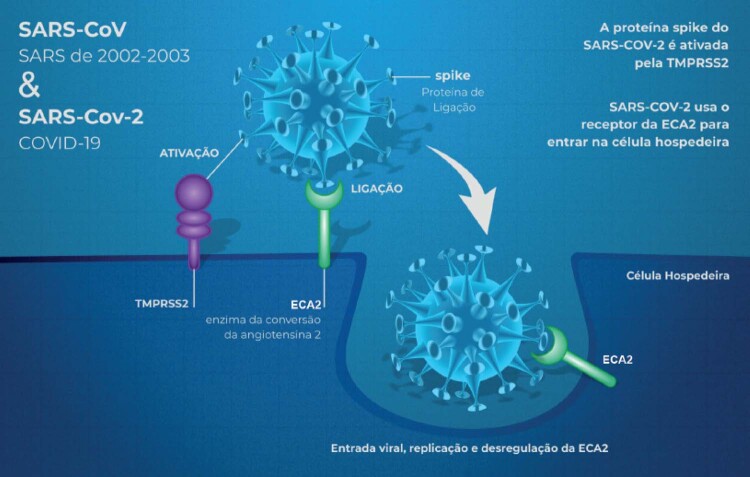
– *O SARS-CoV-2 liga-se por meio da proteína spike da superfície viral ao receptor da ECA-2 humana após a ativação da proteína spike pela TMPRSS2. SARS-CoV: coronavírus da síndrome respiratória aguda grave; SARS-COV-2: coronavírus da síndrome respiratória aguda grave 2; COVID-19: doença do coronavírus 2019; ECA-2: enzima conversora de angiotensina-2; TMPRSS2: serina protease transmembrana-2. Fonte: Costa IBSS, Brittar CS, Rizk SI, et al., 2020.*

A ECA-2 é expressa no pulmão, principalmente nas células alveolares do tipo II, e parece ser o portal de entrada predominante.^2 - 4^ Ao se ligar à ECA-2, SARS-CoV-2 gera *downregulation* desta enzima e determina aumento dos níveis de angiotensina II, o que pode levar aos efeitos deletérios da ativação do sistema renina-angiotensina-aldosterona, tais como vaso constrição, alteração de permeabilidade vascular, remodelamento miocárdico e injúria pulmonar aguda; isto pode justificar, em parte, os sintomas pulmonares frequentes na síndrome^5^ ( Figura 1 ).^6^

A ECA-2 também é altamente expressa no coração, neutralizando os efeitos da angiotensina II em estados com ativação excessiva do sistema renina-angiotensina, como hipertensão arterial sistêmica (HAS), insuficiência cardíaca (IC) e aterosclerose, por converter angiotensina II em angiotensina I-VII, que tem efeito cardioprotetor.

Além do coração e do pulmão, a ECA-2 é expressa no epitélio intestinal, endotélio vascular e rins, fornecendo um mecanismo para a disfunção de múltiplos órgãos que pode ser observada na infecção por SARS-CoV-2.

## COVID-19 e Lesão Miocárdica

A presença de troponina elevada na admissão hospitalar esteve associada com maior mortalidade em dois estudos envolvendo pacientes internados com COVID-19.^7 - 8^

Um destes estudos, desenvolvido em um hospital da Universidade de Wuhan, avaliou uma coorte de 416 pacientes hospitalizados por COVID-19 em que a média de idade foi 64 anos, 50% do sexo feminino e a DCV mais frequente foi HAS (30,5%). Dentre os pacientes incluídos, 82 (19,7%) tiveram injúria miocárdica, definida através da troponina I de alta sensibilidade acima do percentil 99. Pacientes hipertensos tiveram mais injúria miocárdica que aqueles sem hipertensão (59% x 23%); assim como pacientes com doença arterial coronária (DAC) (29,3% x 6,0%); doença cerebrovascular (15,9% x 2,7%) e IC (14,6% x 1,5%) (p < 0,001 para todas as variáveis). Os autores observaram maior frequência de síndrome da angústia respiratória aguda (58,5% x 14,7%, p < 0,001) assim como maior mortalidade entre os pacientes com injúria miocárdica (51% x 4,5%, p < 0,001).^7^

O outro estudo trata-se de uma coorte retrospectiva unicêntrica que avaliou 187 pacientes com COVID-19. A média de idade foi de 58 anos; 35% apresentavam alguma DCV (HAS, DAC ou cardiomiopatia) e 43 pacientes evoluíram a óbito (23%). Os autores observaram troponina T elevada em 27,8% dos casos. A taxa de mortalidade foi em torno de 7% para pacientes sem DCV e troponina T negativa, porém este valor foi 10 vezes maior quando a presença de DCV associou-se à presença de injúria cardíaca.^7^ Vale destacar que os pacientes com DCV que apresentavam troponina T negativa durante a infecção não tiveram mortalidade tão expressiva (13,3%) quanto aqueles que apresentaram elevação de troponina.^8^

Pacientes com troponina elevada eram mais idosos; tinham mais comorbidades e níveis mais elevados de leucócitos, NT-pró-BNP, proteína C-reativa e procalcitonina, mas com contagem mais baixa de linfócitos.

Um estudo demonstrou que, no quarto dia após o início dos sintomas, os níveis médios de troponina foram de 8,8 pg/mL em não sobreviventes vs. 2,5 pg/mL em sobreviventes. Durante o acompanhamento, a mediana de troponina entre os sobreviventes não mudou significativamente (2,5 – 4,4 pg/mL), enquanto subiu para 24,7 pg/mL no sétimo dia, 55,7 pg/mL no décimo terceiro dia, 134,5 pg/mL no décimo nono dia e 290,6 pg/mL no vigésimo segundo dia entre os não sobreviventes. O tempo médio até a morte desde o início dos sintomas foi de 18,5 dias (IQR 15 – 20 dias).^9^

O aumento da troponina foi acompanhado pela elevação de outros biomarcadores inflamatórios (dímero D, ferritina, interleucina-6 [IL-6], desidrogenase lactato), aumentando a possibilidade de que isso reflita mais a tempestade de citocinas ou a linfohistiocitose hematofagocítica secundária do que lesão miocárdica isolada.

## Mecanismos da lesão miocárdica e COVID-19

**Figura 2:**
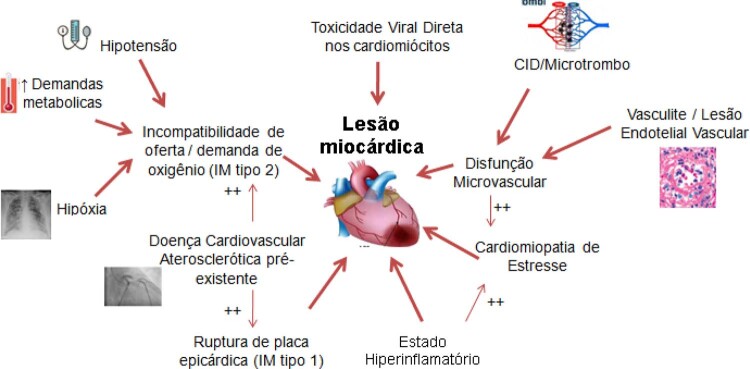
Mecanismos potenciais de lesão miocárdica na COVID-19. IM: infarto do miocárdio; CID: coagulação intravascular disseminada. Fonte: Figura adaptada de Atri D, Siddidi HK, Lang J, et al. COVID-19 for the Cardiologist: A Current Review of the Virology, Clinical Epidemiology, Cardiac and Other Clinical Manifestations and Potential Therapeutic Strategies. JACC Basic Transl Sci. 2020 Apr 10. doi: 10.1016/j.jacbts.2020.04.002. [Epub ahead of print]

Os mecanismos da lesão miocárdica não estão bem estabelecidos, mas provavelmente envolvem aumento do estresse cardíaco devido à insuficiência respiratória e hipoxemia, síndrome coronariana aguda (SCA), lesão indireta da resposta inflamatória sistêmica, infecção miocárdica direta por SARS-CoV-2, entre outros fatores ( Figura 2 ).^10^

### Lesão miocárdica secundária ao desequilíbrio entre oferta e demanda de oxigênio

Situações de grave estresse fisiológico como sepse e insuficiência respiratória presentes em pacientes com COVID-19 estão associadas a elevações de biomarcadores de lesão miocárdica, determinando pior prognostico em alguns pacientes.^11^

O mecanismo mais provável é um desequilíbrio entre oferta e demanda de oxigênio, sem ruptura da placa ateromatosa e consistente com o diagnóstico de infarto do miocárdio tipo 2.^12 , 13^

Estes pacientes têm taxas de mortalidade mais altas, quando comparados com os que apresentam infarto do miocárdio tipo 1, provavelmente decorrente de um maior número de comorbidades.^14^

Devido à idade e ao perfil de comorbidades dos pacientes hospitalizados com COVID-19 grave, pode-se supor que essa população tenha um maior risco de DAC não obstrutiva subjacente e que a ocorrência de infarto do miocárdio tipo 2 contribui para a elevação da troponina e para piores desfechos.^7^

### Lesão microvascular

O provável mecanismo da lesão miocárdica decorre da formação de microtrombos na vasculatura do miocárdio, na presença de um estado de hipercoagulabilidade como na coagulação intravascular disseminada (CIVD). Alterações nos sistemas de coagulação e fibrinolítico estão presentes de maneira importante em pacientes com COVID-19, observando-se CIVD na maioria dos pacientes que faleceram.^15^

Os mecanismos da CIVD no contexto de sepse e síndrome do desconforto respiratório agudo presente nestes pacientes são complexos, mas acredita-se que esteja relacionado a uma exaustão dos sistemas de coagulação e fibrinolítico determinando sangramento e trombose no mesmo paciente.^16^

O aumento das citocinas inflamatórias, como IL-6 e fator de necrose tumoral-alfa (TNF-α), bem como a lesão endotelial, aumentam a expressão do fator tecidual, determinado um estado pró-trombótico.^17^

Por outro lado, a desregulação da antitrombina III, do inibidor do ativador do plasminogênio tipo 1 (PAI-1) e da proteína C em situações de inflamação e sepse significativas promove um estado de anticoagulação.^18^

Além disso, a ativação plaquetária também ocorre no contexto de sepse e inflamação, alterando o delicado equilíbrio do sistema de coagulação.^19^

Desta forma, a presença de inflamação e da ativação imune presentes na infecção grave por COVID-19 podem determinar CIVD, disfunção microvascular e lesão miocárdica.

### Resposta inflamatória sistêmica

Um dos prováveis mecanismos relacionados à lesão cardíaca em pacientes com COVID-19 grave envolve a intensa resposta inflamatória sistêmica. Relatos iniciais demonstraram que níveis extremamente elevados de biomarcadores inflamatórios e citocinas, incluindo IL-6, proteína C-reativa , TNF-α, interleucina-2R (IL-2R) e ferritina estiveram associados a manifestações mais graves de COVID-19 e a piores desfechos.^20^

Vários estudos já demonstraram que a cardiomiopatia na sepse é parcialmente mediada por citocinas inflamatórias como TNF-α, IL-6, IL-1β, INF-γ e IL-2.^21 , 22^

Cardiomiócitos de ratos cultivados demonstraram contratilidade reduzida quando expostos a IL-6. O mecanismo pode ser através da modulação da atividade do canal de cálcio com disfunção miocárdica resultante.^23^

Além disso, acredita-se que o óxido nítrico seja um mediador da depressão do miocárdio em estados de intensa inflamação, como a sepse.^24^

Mais recentemente, a observação do papel da disfunção mitocondrial nos estados sépticos levantou questões sobre o papel dessa entidade na cardiomiopatia associada à sepse.^25^

Pacientes com as formas mais graves de COVID-19 apresentam disfunção multiorgânica com tempestade de citocinas e desregulação imunológica, sendo estes prováveis mecanismos envolvidos na lesão miocárdica observada nestes pacientes.^26^

### Cardiomiopatia por estresse

O papel da cardiomiopatia por estresse (takotsubo) na lesão cardíaca relacionada à COVID-19 ainda não é bem conhecido, com poucos relatos até o momento.^27 - 29^

No entanto, acredita-se que vários dos mecanismos propostos para lesão cardíaca relacionada à COVID-19, detalhados nesta revisão, estejam implicados na fisiopatologia da cardiomiopatia por estresse, particularmente os de disfunção microvascular, tempestade de citocinas e aumento simpático.^30^

O intenso estresse emocional e as infecções respiratórias causadas pela COVID-19 podem representar potenciais gatilhos nesse contexto. É possível que cardiomiopatia por estresse também possa desempenhar um papel significativo na pandemia da COVID-19.

### Síndrome coronariana aguda não obstrutiva

Pacientes com COVID-19 podem apresentar sinais e sintomas clássicos para SCA, tais como dor torácica, alterações eletrocardiográficas sugestivas de isquemia miocárdica ou infarto agudo do miocárdio, tornando difícil este diagnostico diferencial.^31^

Os dados publicados até agora não explicitam a incidência de SCA por ruptura da placa epicárdica, como mecanismo para a lesão cardíaca observada na COVID-19.

Contudo, já existe um conhecimento adquirido, que demonstra a associação entre infecção e um risco elevado de SCA. Estudos epidemiológicos demonstraram que a hospitalização por pneumonia está associada a um maior risco de eventos ateroscleróticos.^32^

Estudos avaliando a infecção por influenza demonstraram associação temporal entre complicações cardiovasculares e SCA, e a vacinação anual contra influenza esteve associada à redução de 36% de eventos cardiovasculares adversos maiores, em metanálise de ensaios clínicos que avaliaram essa questão.^33 , 34^

Desta forma, a infecção viral está associada a um risco aumentado de eventos coronarianos e a prevenção está associada com uma redução desse risco. Portanto, é plausível que a SCA também seja uma causa importante de lesão cardíaca aguda em pacientes com COVID-19. Existem vários possíveis mecanismos fisiopatológicos, pelos quais a infecção viral sistêmica (por influenza ou SARS-CoV-2, por exemplo) pode levar a um maior risco de desestabilização da placa e SCA. Entre eles, o papel da inflamação no desenvolvimento e progressão da aterosclerose está bem estabelecido.^35 - 38^

A resposta imune à infecção viral aguda e o aumento concomitante de citocinas e mediadores inflamatórios presentes na COVID-19 podem levar à inflamação arterial localizada, que pode ser mais pronunciada nas placas coronárias.^39^

A entrada de produtos virais na circulação sistêmica, também conhecidos como padrões moleculares associados a patógenos (PMAP), pode causar a ativação inata do receptor imune, levando à ativação de células imunes residentes em ateroma preexistente, podendo determinar ruptura da placa; além do fato de que os PMAP virais podem ativar o inflamassoma, promovendo a conversão de pró-citocinas nas citocinas biologicamente ativas.^40 , 41^

Por fim, a disfunção endotelial decorrente da infecção e inflamação pode determinar vasoconstricção, com diminuição do fluxo coronariano.^42^

Todas estas alterações fisiopatológicas presentes na COVID-19 podem determinar à desestabilização de placa aterosclerótica pré-existente deflagrando um evento coronariano agudo.

### Lesão miocárdica viral direta

Relatos de casos de miocardite na COVID-19 fornecem evidências de inflamação cardíaca, mas não determinam o mecanismo.

Um dos mecanismos propostos para a lesão miocárdica observada na COVID-19 seria a infecção viral direta do coração, com miocardite resultante.

De fato, o miocárdio humano expressa o receptor utilizado pela COVID-19 para infectar as células hospedeiras, a ECA-2. Assim, sem dúvida, em alguns casos, uma miocardite viral devido a esse agente pode ocorrer.

No entanto, o aumento da troponina parece quase onipresente em pacientes que necessitam de tratamento intensivo, uma indicação de envolvimento cardíaco, que em muitos casos é um marcador de mau prognóstico, como em muitas outras circunstâncias.^41^

Um modelo murino de infecção pulmonar, demonstrada com SARS-CoV-1 também precipitou infecção miocárdica dependente de ECA-2^42 - 43^ . Entre os seres humanos, durante o surto de SARS de Toronto, o RNA do vírus SARS-CoV-1 foi detectado em 35% dos corações autopsiados.^1^ Isso aumenta a possibilidade de danos diretos de cardiomiócitos pelo vírus.^44^

À luz do receptor de entrada da célula hospedeira compartilhado entre SARS-CoV-1 e SARS-CoV-2, uma entrada miocárdica viral direta e a lesão resultante são plausíveis também com SARS-CoV-2. O SARS-CoV-2 pode compartilhar o mesmo mecanismo com o SARS-CoV-1, porque os dois vírus são altamente homólogos no genoma.^45 , 46^

Temos até o momento apenas um relato de miocardite viral por SARS-CoV-2 comprovada por biópsia com inclusões virais ou DNA viral detectado no tecido do miocárdio.^46^ Porem não havia a presença de partículas virais no cardiomiócito, apenas no interior dos macrófagos no interstício cardíaco.

Outro mecanismo hipotético de lesão viral direta ao miocárdio é através de uma vasculite mediada por infecção. O receptor ECA-2 é altamente expresso em artérias e veias endoteliais.^47^

Existem dados patológicos do SARS-CoV-1, mostrando evidências de vasculite com infiltração de monócitos e linfócitos e lesão de células endoteliais no coração.^48^

A entrada viral direta nas células endoteliais do miocárdio pode desencadear uma vasculite, ou a presença do vírus pode levar a uma resposta imunológica indireta e consequente reação de hipersensibilidade.^49 , 50^ Esse insulto estaria associado à lesão miocárdica e talvez até à disfunção miocárdica evidente na COVID-19.

Embora a ECA-2 seja apenas levemente expressa no cardiomiócito, ela é altamente expressa nos pericitos. A COVID-19 pode atacar pericitos essenciais para a estabilidade endotelial, causando disfunção endotelial, que leva a distúrbios microcirculatórios. Isso explica por que, embora a ECA-2 seja apenas ligeiramente expresso nos cardiomiócitos, COVID-19 pode causar lesão cardíaca.^51^

As autópsias mostram infiltrados inflamatórios compostos por macrófagos e, em menor grau, por células T e CD4+.^52 , 53^

Esses infiltrados mononucleares estão associados a regiões de necrose de cardiomiócitos que, pelos critérios de Dallas, definem miocardite.^54^

As análises de PCR em tempo real de tecido cardíaco post mortem da epidemia de SARS detectaram o genoma viral em 35% dos pacientes que morreram de SARS. É importante notar que esses corações também apresentaram níveis diminuídos de ECA-2 e aumento da hipertrofia.^44^

Observados estes dados em conjunto, ainda não está claro quanto da lesão cardíaca é atribuível à infecção viral direta versus toxicidade indireta pela infecção sistêmica. Além disso, não estão definidas quais populações celulares no miocárdio são mais vulneráveis a infecções e/ou inflamação sistêmica. Os níveis de expressão da ECA-2 podem ser importantes, mas novamente as implicações de tais diferenças são discutíveis.

Inciardi et al.,^55^ descrevem uma paciente com COVID-19, que se apresentou com fadiga, aumento da troponina, elevação de peptídeo natriurético tipo B (BNP), alterações eletrocardiográficas, alterações de contração segmentar, derrame pericárdico e disfunção ventricular esquerda ao ecocardiograma com angiotomografia das artérias coronárias normal aproximadamente uma semana após ter apresentado quadro de febre e tosse seca; a ressonância magnética demonstrou acentuado edema intersticial miocárdico biventricular e o padrão de realce tardio do gadolínio sugerindo o diagnóstico de miocardite. A paciente necessitou suporte inotrópico e apresentou melhora clínica e laboratorial a partir de uma semana de tratamento.

Hu et al.,^56^ descreveram um paciente com quadro de dor torácica e dispneia durante três dias, elevação da troponina e BNP; alterações eletrocardiográficas; alterações de contração segmentar; derrame pericárdico e disfunção ventricular esquerda ao ecocardiograma com angiotomografia das artérias coronárias normal. À admissão, apresentava-se hipotenso com quadro sugestivo de miocardite fulminante. Foi tratado com suporte hemodinâmico (drogas vasopressoras e inotrópicas) e metilprednisolona associada à imunoglobulina humana. Após três semanas de tratamento evoluiu com recuperação completa da função ventricular e normalização dos marcadores de lesão miocárdica.

Em suma, nos parece clara a associação entre a presença de injúria miocárdica, identificado através da elevação de troponina, e pior prognóstico em pacientes com COVID-19. Em relação ao diagnóstico de miocardite, definido pela elevação de marcadores, associada a quadro clínico sugestivo, com alterações compatíveis em exames de cardioimagem, foram descritos alguns relatos de casos em pacientes com COVID-19, porém sem dados de biopsia comprovando a causa da miocardite.

Desta forma, considerando que SARS-CoV-1 e SARS-CoV-2 infectam as células através da ECA-2, proteína de membrana presente em células miocárdicas, é possível que esse mecanismo seja responsável também por miocardite em pacientes com diagnóstico de COVID-19. No entanto, maiores evidências são necessárias para comprovar tal associação.

## Conclusão

O envolvimento miocárdico e pericárdico (derrames/pericardite) é comum nas fases graves na doença causada pela COVID-19. O acometimento agudo do miocárdio tem sido descrito como uma injúria cardíaca aguda, induzida por uma possível “tempestade de citoquinas inflamatórias”, podendo ou não ocasionar necrose do cardiomiócito.

Raros casos de infiltrado inflamatório leve e a presença do vírus nas células inflamatórias do interstício cardíaco e nas células endoteliais da microcirculação coronária foram precisamente descritos, confirmando a real presença histológica de miocardite viral, porém até o momento não se descreveu o coronavírus no interior do cardiomiócito. O estado de resposta adrenérgica e inflamação miocárdica pode explicar a ocorrência do padrão fenotípico de síndrome de takotsubo.

Em resumo, o elevado grau de suspeita clínica com dor torácica, alteração hemodinâmica e/ou alterações do ST e/ou arritmias no ECG, associadas a anormalidades morfofuncionais nos métodos de cardioimagem e elevação da troponina cardíaca, representam os pilares do raciocínio clínico para a presença da agressão miocárdica aguda na atual pandemia por coronavírus.

Adicionalmente, torna-se necessário a cardiovigilância destes pacientes, posto que, à luz dos conhecimentos atuais, não sabemos se eles poderão ou não evoluir com disfunção miocárdica tardia.
