# Supplementary material for: Doença de Coronavírus-19 e o Miocárdio
Source: Arq Bras Cardiol. 2020 Jun 29;114(6):1051–7. [Article in Portuguese] doi: 10.36660/abc.20200373 (PMC8416129; doi:10.36660/abc.20200373)
Supplement: Translation

# Translation: Coronavirus Disease 2019 and the Myocardium

**Authors:** José Albuquerque de Figueiredo, Fabiana G. Marcondes-Braga, Lidia Zytinski Moura, André Melo e Silva de Figueiredo, Viviane Melo e Silva de Figueiredo, Ricardo Mourilhe-Rocha, Evandro Tinoco Mesquita

**Affiliations:** 1 Brazil Universidade Federal do Maranhão, São Luis, MA - Brazil; 2 Brazil Instituto do Coração do Hospital das Clínicas da Faculdade de Medicina da Universidade de São Paulo, São Paulo, SP - Brazil; 3 Brazil Pontifícia Universidade Católica do Paraná, Curitiba, PR - Brazil; 4 Brazil Universidade do Estado do Rio de Janeiro e Hospital Pró-cardíaco. Brazil; 5 Brazil Educador C.T.E.B./UHG.Brazil

**Keywords:** Myocardium/injuries, Troponin, Inflammatory Diseases, Myocarditis, Takotsubo Syndrome, Biomarkers, Coronavirus, COVID-19, Pandemics, Cardiomyopathy, Dilated, Thrombotic Microangiopathies

## Abstract

Infection with the coronavirus known as COVID-19 has promoted growing interest on the part of cardiologists, emergency care specialists, intensive care specialists, and researchers, due to the study of myocardial involvement based on different clinical forms resulting from immunoinflammatory and neurohumoral demodulation.Myocardial involvement may be minimal and identifiable only by electrocardiographic changes, mainly increased cardiac troponins, or, on the other side of the spectrum, by forms of fulminant myocarditis and takotsubo syndrome.The description of probable acute myocarditis has been widely supported by the observation of increased troponin in association with dysfunction. Classical definition of myocarditis, supported by endomyocardial biopsy of inflammatory infiltrate, is rare; it has been observed in only one case report to date, and the virus has not been identified inside cardiomyocytes.Thus, the phenomenon that has been documented is acute myocardial injury, making it necessary to rule our obstructive coronary disease based on increased markers of myocardial necrosis, whether or not they are associated with ventricular dysfunction, likely associated with cytokine storms and other factors that may synergistically promote myocardial injury, such as sympathetic hyperactivation, hypoxemia, arterial hypotension, and microvascular thrombotic phenomena.Systemic inflammatory and myocardial phenomena following viral infection have been well documented, and they may progress to cardiac remodeling and myocardial dysfunction. Cardiac monitoring of these patients is, therefore, important in order to monitor the development of the phenotype of dilated myocardiopathy.This review presents the main etiological and physiopathological findings, a description of the taxonomy of these types of cardiac involvement, and their correlation with the main clinical forms of the myocardial component present in patients in the acute phase of COVID-19.

## Introduction

Myocardial injury, as shown by increased cardiac biomarkers, was identified among the first cases of COVID-19 in China. The National Health Council of China reported that almost 12% of patients without known cardiovascular disease (CVD) showed elevated levels of troponin or cardiac arrest during hospitalization.^1^

These findings have stimulated research and interest on the part of cardiologists, intensive care specialists, and clinical researchers, due to early recognition of these abnormalities, as well as the search for physiopathological mechanisms and their real impacts on prognosis.

In addition to this, individuals with previous CVD have been shown to be at a higher risk of developing severe forms and higher mortality.

Accordingly, it is of fundamental importance to understand the spectrum of myocardial involvement, whether primary or secondary, in addition to the etiological and physiopathological mechanisms involved, in order to promote the development of therapeutic strategies that can prevent and diminish myocardial aggression during the acute phase.

## SARS-CoV-2 and the mechanism of direct cellular aggression

SARS-CoV-2 infection is caused by binding of the spike protein on the surface of the virus to the human angiotensin converting enzyme 2 (ACE-2) receptor after activation of the spike protein by transmembrane protease, serine 2 (TMPRSS2).

ACE-2 is expressed in the lungs, mainly in the type-II alveolar cells, and it seems to be the predominant means of entry.^2 - 4^ SARS-CoV-2, in binding to ACE-2, causes downregulation of this enzyme, determining an increase in levels of angiotensin II, which may lead to deleterious effects in the activation of the renin-angiotensin-aldosterone system, such as vessel constriction, changes in vascular permeability, myocardial remodeling, and acute pulmonary injury, which may partially justify the frequent pulmonary symptoms in this syndrome^5^ ( Figure 1 ).^6^

**Figure 1:** – *By means of its surface spike protein, SARS-CoV-2 binds to the human ACE-2 receptor following activation of the spike protein by TMPRSS2. SARS-CoV: severe acute respiratory syndrome coronavirus; SARS-COV-2: severe acute respiratory syndrome coronavirus 2; COVID-19: coronavirus disease 2019; ACE-2: angiotensin-converting enzyme-2; TMPRSS2: transmembrane protease, serine 2. Source: Costa IBSS, Brittar CS, Rizk SI, et al., 2020.*

ACE-2 is also highly expressed in the heart, neutralizing the effects of angiotensin II in states with excessive activation of the renin-angiotensin system, such as systemic arterial hypertension (SAH), heart failure (HF), and atherosclerosis, by converting angiotensin II into angiotensin I-VII, which has a cardioprotective effect.

In addition to the heart and lungs, ACE-2 is expressed in the intestinal epithelium, vascular endothelium, and kidneys, providing a mechanism for multiple organ dysfunction, which can be observed in SARS-CoV-2 infection.

## 3- COVID-19 and Myocardial Injury

Increased troponin upon admission to the hospital has been associated with higher mortality in two studies involving patients hospitalized with COVID-19.^7 - 8^

One of these studies, which was conducted in a hospital at Wuhan University, evaluated a cohort of 416 patients hospitalized for COVID-19, with a mean age of 64 years, 50% of whom were female; the most frequent CVD was SAH (30.5%). Of the patients included, 82 (19.7%) had myocardial injury, defined as high-sensitivity troponin I above the 99th percentile. Patients with hypertension had more myocardial injury than those without hypertension (59% vs. 23%). The same was the case patients for patients with coronary artery disease (CAD) (29.3% vs. 6.0%); cerebrovascular disease (15.9% vs. 2.7%), and HF (14.6% vs. 1.5%) (p < 0.001 for all variables). The authors observed greater frequency of acute respiratory distress syndrome (58.5% vs. 14.7%, p < 0.001) and greater mortality among patients with myocardial injury (51% x 4.5%, p < 0.001).^7^

The second, a single-center retrospective study, evaluated a cohort of 187 patients with COVID-19. Mean age was 58 years; 35% had some CVD (SAH, CAD, or cardiomyopathy), and 43 patients progressed to death (23%). The authors observed increased troponin T in 27.8% of cases. Mortality rate was around 7% for patients without CVD and negative troponin T; this value was ten-fold when the presence of CVD was associated with the presence of cardiac injury.^7^ It is worth underscoring that mortality in patients with CVD, who nonetheless had negative troponin T during infection, was not as expressive (13.3%) as mortality in those with increased troponin.^8^

Patients with increased troponin were more elderly. They had more comorbidities; higher levels of leukocytes, NT-pro-BNP, C-reactive protein, and procalcitonin; and lower lymphocyte counts.

Another study demonstrated that, on the fourth day after onset of symptoms, mean troponin levels were 8.8 pg/mL in patients who did not survive, in comparison with 2.5 pg/mL in those who survived. During follow-up, median troponin among survivors did not change significantly (2.5 – 4.4 pg/mL), while it rose to 24.7 pg/mL on the seventh day, 55.7 pg/mL on the thirteenth day, 134.5 pg/mL on the nineteenth day, and 290.6 pg/mL on the twenty-second day among patients who did not survive. Average time to death after onset of symptoms was 18.5 days (IQR 15 - 20 days).^9^

The increase in troponin was accompanied by an increase in other inflammatory biomarkers (D dimer, ferritin, interleukin-6 (IL-6), and lactate dehydrogenase), thus increasing the chance that this reflects a cytokine storm or secondary hemophagocytic lymphohistiocytosis, rather than isolated myocardial injury.

## Mechanisms of myocardial injury and COVID -19

The mechanisms of myocardial injury are not well established, but they probably involve an increase in cardiac stress due to respiratory failure and hypoxemia, acute coronary syndrome (ACS), indirect lesion from the systemic inflammatory response, direct myocardial infection by SARS-CoV-2, and other factors ( Figure 2 ).^10^

**Figure 2:** – *Potential mechanisms of myocardial injury in COVID-19. DIC: disseminated intravascular coagulation; MI: myocardial infarction. Source: Atri D, Siddidi HK, Lang J, et al. COVID-19 for the Cardiologist: A Current Review of the Virology, Clinical Epidemiology, Cardiac and Other Clinical Manifestations and Potential Therapeutic Strategies. JACC Basic Transl Sci. 2020 Apr 10. doi: 10.1016/j.jacbts.2020.04.002. [Epub ahead of print]*

### Myocardial injury secondary to imbalance between oxygen supply and demand

Situations of severe physiological stress, such as sepsis and respiratory failure, which are present in patients with COVID-19, are associated with increased biomarkers of myocardial injury, leading to worse prognosis in some patients.^11^

The most likely mechanism is an imbalance between oxygen supply and demand, without rupture of the atheromatous plaque, consistent with diagnosis of type 2 myocardial infarction.^12 , 13^

These patients have higher rates of mortality when compared to those with type 1 myocardial infarction, likely as a result of a greater number of comorbities.^14^

Due to age and the comorbidity profile of patients hospitalized with severe COVID-19, it may be inferred that this population has a higher risk of underlying non-obstructive CAD and that the occurrence of type 2 myocardial contributes to increased troponin and worse outcomes.^7^

### Microvascular injury

The likely mechanism of myocardial injury results from the formation of microthrombi in the myocardial vasculature, in the presence of a state of hypercoagulability as in disseminated intravascular coagulation (DIC). Changes in the coagulation and fibrinolytic systems are important in patients with COVID-19, and DIC has been observed in the majority patients who died.^15^

The mechanisms of DIC in the context of sepsis and acute respiratory distress syndrome present in these patients are complex; it is believed to be related to an exhaustion of the coagulation and fibrinolytic systems causing both bleeding and thrombosis.^16^

The increase in inflammatory cytokines, such as IL-6 and tumor necrosing factor-alpha (TNF-α), as well as endothelial injury, increase the expression of tissue factor, leading to a pro-thrombotic state.^17^

On the other hand, dysregulation of antithrombin III, plasminogen activator inhibitor type 1 (PAI-1), and protein C in significant situations of inflammation and sepsis promotes a state of anticoagulation.^18^

Furthermore, platelet activation also occurs in the context of sepsis and inflammation, changing the delicate balance of the coagulation system.^19^

In this manner, the presence of inflammation and the immune activation present in severe COVID-19 infection may lead to DIC, microvascular dysfunction, and myocardial injury.

### Systemic inflammatory response

One of the likely mechanisms related to cardiac injury in patients with severe COVID-19 involves the intense systemic inflammatory response. Initial reports demonstrate that extremely high levels of inflammatory biomarkers and cytokines, IL-6, C-reactive protein, TNF-α, interleukin-2R (IL-2R), and ferritin were associated with more severe manifestations of COVID-19 and worst outcomes.^20^

Several studies have demonstrated that cardiomyopathy in sepsis is partially mediated by inflammatory cytokines such as TNF-α, IL-6, IL-1β, INF-γ, and IL-2.^21 , 22^

Cultivated rat cardiomyocytes demonstrated reduced contractility when exposed to IL-6. The mechanism may be through modulated calcium channel activity with resulting myocardial dysfunction.^23^

It is furthermore believed that nitric oxide is a mediator of myocardial depression in states of intense inflammation, such as sepsis.^24^

More recently, observation of the role of mitochondrial dysfunction in septic states raised questions concerning the role of this entity in cardiomyopathy associated with sepsis.^25^

Patients with more severe forms of COVID-19 have multiple organ dysfunction with cytokine storms and immune dysregulation, which are likely mechanisms involved in the myocardial injury observed in these patients.^26^

### Stress cardiomyopathy

The role of stress cardiomyopathy (takotsubo syndrome) in cardiac injury related to COVID-19 is still not well known, and there are few reports to date.^27 - 29^

It is, however, believed that several of the proposed mechanisms for cardiac injury related to COVID-19 that are detailed in this review are implicated in the pathophysiology of stress cardiomyopathy, especially microvascular dysfunction, cytokine storm, and sympathetic increase.^30^

The intense emotional stress and the respiratory infections caused by COVID-19 may represent potential triggers in this context. It is possible that stress cardiomyopathy may also play a significant role in the COVID-19 pandemic.

### Non-obstructive acute coronary syndrome

Patients with COVID-19 may have more classical signs and symptoms of ACS, such as chest pain and electrocardiographic changes suggestive of myocardial ischemia or acute myocardial infarction, making this differential diagnosis difficult.^31^

The data published to date do not explain the incidence of ACS due to epicardial plaque rupture, as a mechanism for the cardiac injury observed in COVID-19.

Nonetheless, existing acquired knowledge demonstrates the association between infection and increased risk of ACS. Epidemiological studies have demonstrated that hospitalization due to pneumonia is associated with increased risk of atherosclerotic events.^32^

Studies evaluating influenza infection have demonstrated a temporal association between cardiovascular complications and ACS, and annual vaccination against influenza was associated with a 36% decrease in major adverse cardiovascular events in a meta-analysis of clinical trials evaluating this question.^33 , 34^

In this manner, viral infection is associated with an increased risk of coronary events, and prevention is associated with reduced risk. It is, therefore, plausible that ACS is also an important cause of acute cardiac injury in patients with COVID-19. There are several possible pathophysiological mechanisms whereby systemic viral infection (by influenza or SARS-CoV-2, for example) can lead to an increased risk of plaque destabilization and ACS. The role of inflammation in the development and progression of atherosclerosis is well established.^35 - 38^

The immune response to acute viral infection and the concomitant increase in cytokines and inflammatory mediators present in COVID-19 can lead to localized arterial inflammation, which may be more pronounced in coronary plaque.^39^

The entrance of viral products into systemic circulation, also known as pathogen-associated molecular patterns (PAMP), can lead to innate activation of the immune receptor, in turn leading to activation of immune cells residing in pre-existing atheroma, which may cause plaque rupture; furthermore, viral PAMP can activate the inflammasome, promoting conversion of pro-cytokines to biologically active cytokines.^40 , 41^

Finally, endothelial dysfunction resulting from infection and inflammation may lead to vessel constriction, with decreased coronary flow.^42^

All of these physiopathological alterations present in COVID-19 can lead to destabilization of pre-existing atherosclerotic plaque, thus triggering an acute coronary event.

### Direct viral myocardial injury

Reports of cases of myocarditis in COVID-19 provide evidence of cardiac inflammation, but they do not determine the mechanism.

One of the proposed mechanisms behind the myocardial injury observed in COVID-19 is direct viral infection of the heart, with resulting myocarditis.

In fact, the human myocardium expresses the receptor used by COVID-19 to infect host cells, namely, ACE-2. Thus, without a doubt, in some cases, viral myocarditis may occur due to this agent.

The increase in troponin, however, appears to be omnipresent in patients who require intensive care, an indication of cardiac involvement, which is a marker of poor prognosis in many cases, as in many other circumstances.^41^

A murine model of lung infection, demonstrated with SARS-CoV-1, also precipitated myocardial infection dependent on ACE-2^42 - 43^ . In human beings, during the SARS outbreak in Toronto, RNA of the SARS-CoV-1 virus was detected in 35% of autopsied hearts.^1^ This increases the likelihood of direct viral damage to cardiomyocytes.^44^

In view of the host cell input receptor shared by SARS-CoV-1 and SARS-CoV-2, direct viral myocardial entry and the resulting injury is also plausible with SARS-CoV-2. SARS-CoV-2 may share the same mechanism with SARS-CoV-1, given that the two viruses have highly homologous genomes.^45 , 46^

To date, we have only one report of viral myocarditis due to SARS-CoV-2 confirmed by biopsy, with viral inclusions of viral DNA detected in myocardial tissue.^46^ Viral particles, however, were not present in cardiomyocytes, but only inside macrophages in the cardiac interstice.

Another hypothetical mechanism behind direct viral myocardial injury is due to infection-mediated vasculitis. The ACE-2 receptor is highly expressed in arteries and endothelial veins.^47^

There are pathological data on SARS-CoV-1, showing evidence of vasculitis with the infiltration of monocytes and lymphocytes, as well as endothelial cell injury in the heart.^48^

Direct viral entry in endothelial cells of the myocardium can trigger vasculitis, or the presence of the virus can lead to an indirect immunological response and consequent reaction of hypersensitivity.^49 , 50^ This injury would be associated with myocardial injury and perhaps also with the myocardial dysfunction that is evident in COVID-19.

Even though ACE-2 is only slightly expressed in cardiomyocytes, it is highly expressed in pericytes. COVID-19 may attack pericytes, which are essential to endothelial stability, thus causing endotelial dysfunction, which leads to microcirculatory disorders. This explains why COVID-19 may cause cardiac injury, even though ACE-2 is only slightly expressed in cardiomyocytes.^51^

Autopsies have shown inflammatory infiltrates composed of macrophages and, to a lesser extent, T and CD4 + cells.^52 , 53^

These mononuclear infiltrates are associated with areas of cardiomyocyte necrosis, which, according to the Dallas criteria, define myocarditis.^54^

Real-time PCR analyses of post-mortem cardiac tissue from the SARS-CoV-1 epidemic detected the viral genome in 35% of patients who died of SARS-CoV-1. It is important to note that these hearts also showed decreased levels of ACE-2 and increased hypertrophy.^44^

Observing these data together, it is still not clear to what extent cardiac injury is attributable to direct viral infection versus indirect toxicity due to systemic infection. Furthermore, it has yet to be defined which cell populations in the myocardium are most vulnerable to infections and/or systemic inflammation. Levels of expression of ACE-2 may be important, but the implications of such differences are still debatable.

Inciardi et al.^55^ described a patient with COVID-19 who presented with fatigue, increased troponin, increased BNP, electrocardiographic changes, changes in segmental contraction, pericardial effusion, and left ventricular dysfunction on echocardiogram, with normal coronary angiography approximately one week after having presented fever and dry cough; magnetic resonance demonstrated biventricular myocardial interstitial edema, and diffuse late gadolinium enhancement suggesting diagnosis of myocarditis. The patient required inotropic support, and she showed clinical and laboratorial improvement after one week after treatment.

Hu et al.^56^ described a patient with chest pain and dyspnea for three days, as well as increased troponin and BNP, electrocardiographic changes, changes in segmental contraction, pericardial effusion, and left ventricular dysfunction, with normal coronary angiography. Upon admission, he had hypotension with clinical picture suggestive of fulminant myocarditis. He was treated with hemodynamic support (vasopressor and inotropic drugs) and methylprednisolone associated with human immunoglobulin. After three weeks of treatment, the patient evolved with complete recovery of ventricular function and normalized markers of myocardial injury.

In short, it seems clear that there is an association between the presence of myocardial injury, identified by increased troponin, and worse prognosis in patients with COVID-19. In relation to diagnosis of myocarditis, as defined by elevated markers, associated with a suggestive clinical picture and compatible alterations on cardiac imaging exams, some case reports have been described in patients with COVID-19, but without biopsy data confirming the cause of myocarditis.

In this manner, considering that SARS-CoV-1 and SARS-CoV-2 infect cells through ACE-2, a membrane protein present in myocardial cells, it is possible that this mechanism is also responsible for myocarditis in patients diagnosed with COVID-19. However, more evidence is needed to prove this association.

## Conclusion

Myocardial and pericardial involvement (strokes/pericarditis) is common in severe phases of COVID-19. Acute myocardial involvement has been described as acute cardiac injury, induced by a possible “inflammatory cytokine storm,” which may or may not cause cardiomyocyte necrosis.

Rare cases of mild inflammatory infiltrate and the presence of the virus in inflammatory cells of the cardiac interstice and the endothelial cells of coronary microcirculation have been precisely described, confirming the real histological presence of viral myocarditis, but, to date, the coronavirus has not been described inside the cardiomyocyte. The state of adrenergic response and myocardial inflammation may explain the occurrence of the phenotypic pattern of takotsubo syndrome.

In summary, high degree of clinical suspicion, characterized by chest pain, hemodynamic changes and/or changes in ST/Te arrhythmias (ECG), associated with morphofunctional abnormalities in cardiac imaging methods, and increased cardiac troponin, represent the pillars of clinical reasoning for the presence of acute myocardial aggression in the current coronavirus pandemic.

Furthermore, cardiac monitoring has become necessary for these patients, given that, in light of the current knowledge, we do not know whether or not they may progress with late myocardial dysfunction.
